# Prediction of Overall Survival and Progression-Free Survival by the ^18^F-FDG PET/CT Radiomic Features in Patients with Primary Gastric Diffuse Large B-Cell Lymphoma

**DOI:** 10.1155/2019/5963607

**Published:** 2019-10-30

**Authors:** Yi Zhou, Xue-Lei Ma, Lu-Tong Pu, Ruo-Fan Zhou, Xue-Jin Ou, Rong Tian

**Affiliations:** ^1^Department of Nuclear Medicine, West China Hospital, Sichuan University, 37# GuoXueLane, Chengdu 610041, China; ^2^Department of Biotherapy, Cancer Center, State Key Laboratory of Biotherapy, West China Hospital, Sichuan University, 37# GuoXueLane, Chengdu 610041, China; ^3^West China School of Medicine, West China Hospital, Sichuan University, 37# GuoXueLane, Chengdu 610041, China

## Abstract

*Purpos*e. To determine whether the radiomic features of ^18^F-fluorodeoxyglucose (FDG) positron emission tomography/computed tomography (PET/CT) contribute to prognosis prediction in primary gastric diffuse large B-cell lymphoma (PG-DLBCL) patients. *Methods*. This retrospective study included 35 PG-DLBCL patients who underwent PET/CT scans at West China Hospital before curative treatment. The volume of interest (VOI) was drawn around the tumor, and radiomic analysis of the PET and CT images, within the same VOI, was conducted. The metabolic and textural features of PET and CT images were evaluated. Correlations of the extracted features with the overall survival (OS) and progression-free survival (PFS) were evaluated. Univariate and multivariate analyses were conducted to assess the prognostic value of the radiomic parameters. *Results*. In the univariate model, many of the textural features, including kurtosis and volume, extracted from the PET and CT datasets were significantly associated with survival (5 for OS and 7 for PFS (PET); 7 for OS and 14 for PFS (CT)). Multivariate analysis identified kurtosis (hazard ratio (HR): 28.685, 95% confidence interval (CI): 2.067–398.152, *p*=0.012), metabolic tumor volume (MTV) (HR: 26.152, 95% CI: 2.089–327.392, *p*=0.011), and gray-level nonuniformity (GLNU) (HR: 14.642, 95% CI: 2.661–80.549, *p*=0.002) in PET and sphericity (HR: 11.390, 95% CI: 1.360–95.371, *p*=0.025) and kurtosis (HR: 11.791, 95% CI: 1.583–87.808, *p*=0.016), gray-level nonuniformity (GLNU) (HR: 6.934, 95% CI: 1.069–44.981, *p*=0.042), and high gray-level zone emphasis (HGZE) (HR: 9.805, 95% CI: 1.359–70.747, *p*=0.024) in CT as independent prognostic factors. *Conclusion*. ^18^F-FDG PET/CT radiomic features are potentially useful for survival prediction in PG-DLBCL patients. However, studies with larger cohorts are needed to confirm the clinical prognostication of these parameters.

## 1. Introduction

The incidence of extranodal lymphomas has increased steadily over the past 20–30 years, and the most common extranodal site of non-Hodgkin's lymphoma (NHL) is the stomach. Meanwhile, primary gastric lymphoma (PGL) is a rare tumor, and diffuse large B-cell lymphoma (DLBCL) accounts for 59% of cases [1, 2]. The global therapeutic approach to PGL has shifted from surgery to chemotherapy over the past 10 years [2]. With the administration of rituximab in addition to chemotherapy, the outcome of patients with DLBCL has improved from a 45% to 60% 5-year progression-free survival (PFS) [3, 4]. Nevertheless, PG-DLBCL, with nonspecific symptoms, termed “high-grade gastric lymphoma,” has a low complete remission rate and short survival period [1]. The International Prognostic Index (IPI) is currently used for estimating pretreatment risk, though the IPI often does not reliably predict the individual patient outcome because DLBCL tends to behave heterogeneously [5]. Using ^18^F-fluorodeoxyglucose (FDG) positron emission tomography/computed tomography (PET/CT), which depicts the lesion glycolytic activity, several studies have tested the use of metabolic intensity for predicting the PFS and overall survival (OS) of patients with lymphoma [6–8].

The predictive value of PET image analysis for clinical prognosis has been investigated, and the most frequently used parameter is the maximum standardized uptake value (SUV_max_), as it provides an observer-independent measurement [9, 10]. However, many factors can affect the reliability of SUV_max_, such as the decay of the injected dose, the time between injection and imaging acquisition, the partial volume effects, and technological characteristics and parameters [11]. Recently, new metrics derived from staging PET estimating the overall tumor burden, such as the metabolic tumor volume (MTV) or total lesion glycolysis (TLG), have been used to predict PFS and OS in patients with lymphoma [12, 13]. Radiomics, including texture analysis, is a rapidly evolving research field that requires clinicians to extract a large amount of quantitative data from images to assess the intratumoral biological heterogeneity and obtain prognostic information that cannot be acquired visually [14]. Radiomic features can be classified into shape, first-order, second-order, and higher-order features. Shape features describe the shape of the volume of interest (VOI) and its geometric properties such as volume, maximum diameter different orthogonal directions, and sphericity. First-order features, also termed “histogram analysis,” consider the distribution of individual voxel values without concern for spatial relationships, whereas second-order features provide a measure of the spatial arrangement of the voxel intensities and intralesion heterogeneity, such as the gray-level cooccurrence matrix (GLCM) and gray-level run length matrix (GLRLM). Higher-order statistics features are obtained by statistical methods after applying filters or mathematical transforms to the images, for example, suppressing noise or highlighting details to identify repetitive or nonrepetitive patterns. Depending on how the pixels are analyzed, it is possible to extract features of local or regional nature [15]. Moreover, the prognostic information provided by images based on heterogeneity evaluation could lead to more personalized therapy, which may reduce the occurrence of toxicity. In this manner, the possibility of a favorable outcome is increased, and patients at high risk of treatment failure could be provided with intensified therapy regimens [16].

The textural features of ^18^F-FDG PET have been demonstrated to be useful in predicting the outcomes of patients with several types of cancer, including head and neck cancer, esophageal cancer, and non-small-cell lung cancer [17–19]. It is reported that CT-based texture analysis proves to provide prognostic information for patients with Hodgkin's and aggressive non-Hodgkin's lymphomas [20–28]. To our knowledge, no previous study has associated radiomic signatures from either FDG-PET or CT with the outcome of patients with PG-DLBCL. Therefore, our study aims to investigate the prognostic ability of the radiomic features of ^18^F-FDG PET and the low-dose CT component of pretreatment PET-CT in patients with PG-DLBCL.

## 2. Materials and Methods

### 2.1. Patient Population

The study was approved by the institutional ethics review board of the West China Hospital, Sichuan University. Informed consent was waived because this was a retrospective study. In this retrospective single-center investigation, the following inclusion/exclusion criteria were applied to select patients from the institutional database. The inclusion criteria were (a) patients with biopsy-proven PG-DLBCL and (b) those who underwent an FDG-PET/CT scan at baseline at our institution between December 2012 and December 2017. The exclusion criteria were (a) patients with incomplete clinical or imaging datasets and (b) patients with concomitant or previous other cancer types. In total, 35 patients who were treated with the R-CHOP (R-CHOP including cyclophosphamide, doxorubicin, vincristine, prednisone plus rituximab) regimen were included in our study (17 men and 18 women, mean age: 58 years, age range: 26–79 years). For each patient, clinical information (including age, sex, lactate dehydrogenase, B symptoms, Ann Arbor staging, and IPI score), PET-CT images, and follow-up data were acquired. The patients' clinical characteristics are summarized in Table 1.

### 2.2. Image Acquisition

FDG-PET/CT scanning was performed according to the European Association of Nuclear Medicine guidelines version 1.0 and, from February 2015, version 2.0. All images were acquired on a Gemini GXL PET/CT scanner (Philips, Amsterdam). The patients were instructed to fast for ≥6 h, and the blood glucose levels were confirmed to be <200 mg/dL before intravenous administration of ^18^F-FDG approximately 5 MBq/kg body weight (up to 550 MBq). PET/CT scans were carried out approximately 60 min after injection. During image acquisition, a CT scan (120 kVp, 40 mA) with a tube rotation rate of 0.8 s was obtained (the thickness of a section was 4 mm), followed by a PET scan (2 min/bed position, with 5–7 bed positions per patient) without changing the patient's position. Images were reconstructed with standard 4 × 4 × 4 mm^3^ voxels using iterative list mode time-of-flight algorithms, and corrections for attenuation, dead-time, and random and scatter events were applied, without postreconstruction smoothing.

### 2.3. Image Analysis

The VOI in the primary tumor lesion was semiautomatically defined on PET images with a threshold of 40% of the SUV_max_, with segmentation corrections performed manually by consensus by two nuclear medicine-certified physicians. The radiomic analysis was conducted on the PET and CT images within the same VOI. Features were measured using local image features extraction (LIFEx) software. The position of the VOI on the CT images was manually adjusted by consensus to identify the correct position of the lesion when respiratory movements resulted in a mismatch between CT and PET images. Intensity discretization for PET data was performed to reduce the continuous scale to 64 bins with absolute scale bounds between 0 and 20. Similarly, intensity discretization for CT images was performed with the number of gray levels of 400 bins and absolute scale bounds between −1000 and 3000 HU. The parameters calculated from LIFEx reflected the VOI shape, VOI voxel values, histogram of the VOI values, and VOI textural content [29]. The 44 heterogeneous textural features included conventional and histogram-based parameters, shape and size, and second and higher-order features, as detailed in Table 2. Because heterogeneity quantification in PET images using textural features can be confounded by tumor volume effects in small-volume tumor, especially those <10 cm^3^ [30], we only performed these textural analyses for MTVs >10 cm^3^.

### 2.4. Statistical Analysis

The endpoints of this research were OS and PFS. OS was defined as the period from the date of PET/CT image acquisition to the date of death or final follow-up. PFS was defined as the duration between the time of PET/CT image acquisition to the time of disease progression, relapse, death, or final follow-up. The cutoff value of each texture index was defined by the receiver operating characteristic curve according to Youden's index, a value related to the sum of sensitivity and specificity. In addition, the cutoff point was used to stratify high-risk and low-risk groups. Kaplan–Meier analysis was performed to draw survival curves tested by log-rank tests. All clinical characteristics and the radiomic parameters were tested using univariate cox regression analysis. The correlation between these features was evaluated with Spearman's correlation coefficient in order to assess potential redundancy between these features. A threshold of 0.90 was set when testing correlations between features. All uncorrelated predictors identified as significant (*p* < 0.05; *p* values were corrected for false-discovery rate) after multiple testing corrections (with the Benjamini–Hochberg method) were fed into a multivariate cox proportional hazard regression model to identify those independently associated with the survival of PG-DLBCL patients. SPSS version 23.0 (IBM Corporation, Armonk, NY, USA) was used for all statistical analyses.

## 3. Results

### 3.1. Patient Characteristics

The patient characteristics are provided in Table 1. Among 128 PG-DLBCL patients, 93 were excluded due to meeting the exclusion criteria. The study cohort comprised 35 patients with a median age of 58 years (range 26–79 years), including 17 men (48.6%) and 18 women (51.4%). The death occurred in five patients within an average time of 8.2 months (range: 1–14 months) from the baseline PET/CT, and relapse or progression of disease occurred in seven patients within an average time of 21.7 months (range: 1–33). The median OS and PFS were 23.9 and 23.6 months (range: 1–60 months for both), respectively.

### 3.2. Univariate Analysis

A univariate cox regression analysis was performed to evaluate the correlations among the clinicopathological characteristics, textural indices, and survival of the patients. The results of the univariate analysis are provided in Tables 3 and 4. In univariate analyses, MTV (*p*=0.022, *p*=0.013), volume (*p*=0.038, *p*=0.026), coarseness (*p*=0.038, *p*=0.026), and GLNU_GLRLM_ (*p*=0.009, *p*=0.002) were found to be significantly associated with OS and PFS, respectively; kurtosis (*p*=0.022) was found to be significantly associated with OS; and B symptoms (*p*=0.045), compacity (*p*=0.036), and run length nonuniformity (RLNU) (*p*=0.048) were found to be significantly associated with PFS. Regarding the CT parameters, seven texture parameters, including kurtosis (*p*=0.021, *p*=0.039), volume (*p*=0.038, *p*=0.026), GLNU_GLRLM_ (*p*=0.022, *p*=0.013), RLNU_GLRLM_ (*p*=0.038, *p*=0.026), HGZE_GLZLM_ (*p*=0.031, *p*=0.018), long-zone low gray-level emphasis (LZLGE) (*p*=0.032, *p*=0.017), and GLNU_GLZLM_ (*p*=0.038, *p*=0.026) were found to be significantly associated with OS and PFS, respectively. Moreover, B symptoms (*p*=0.045), sphericity (*p*=0.032), high gray-level run emphasis (HGRE) (*p*=0.041), long-run high gray-level emphasis (LRHGE) (*p*=0.040), long-zone emphasis (LZE) (*p*=0.033), long-zone high gray-level emphasis (LZHGE) (*p*=0.033), and zone percentage (*p*=0.034) were found to be significantly associated with PFS, but not with OS. Other texture indices exhibited no significant associations with the survival of PG-DLBCL patients.

### 3.3. Multivariate Analysis

When multivariate cox regression analysis was performed regarding the significant clinicopathological characteristics and textural parameters identified in the univariate analysis, and MTV (hazard ratio (HR): 26.152, 95% confidence interval (CI): 2.089–327.392, *p*=0.011) and kurtosis (HR: 28.685, 95% CI: 2.067–398.152, *p*=0.012) were the independent predictors of OS, while GLNU_GLRLM_ (HR: 14.642, 95% CI: 2.661–80.549, *p*=0.002) was an independent predictor of PFS. Regarding the CT parameters, kurtosis (HR: 11.791, 95% CI: 1.583–87.808, *p*=0.016) and HGZE_GLZLM_ (HR: 9.805, 95% CI: 1.359–70.747, *p*=0.024) were regarded as independent predictors of OS. Moreover, sphericity (HR: 11.390, 95% CI: 1.360–95.371, *p*=0.025), GLNU_GLZLM_ (HR: 6.934, 95% CI: 1.069–44.981, *p*=0.042), and HGZE_GLZLM_ (HR 11.504, 95% CI 1.921–68.888, *p*=0.007) were regarded as independent predictors of PFS. The results of the multivariate analysis are summarized in Tables 5 and 6.

## 4. Discussion

In our study, we assessed the utility of a radiomic approach in outcome prediction in PG-DLBCL patients. Our results suggest that five textural parameters, including MTV, kurtosis, and HGZE_GLZLM_, are independent parameters that can be used to predict the survival of patients with PG-DLBCL.

^18^F-FDG PET/CT, a whole-body metabolic imaging technique, plays an important role in the staging, treatment monitoring, and prognostication assessment of lymphoma [8]. Furthermore, the predictive value of ^18^F-FDG PET/CT image analysis for clinical prognosis has also been investigated [31–33]. Due to the stability and reproductivity, SUV_max_ has been the most frequently used parameter in previous reports [20] despite some limitations as mentioned before and, additionally, the unestablished prognostic role. Despite the correlation between SUV_max_ and survival, our results, consistent with previous studies, confirmed the absence of such a relationship for OS and PFS [34, 35]; some studies have suggested a correlation between the SUV_max_ and survival [36–38]. The reason for this discrepancy may be due to the fact that SUV_max_ reflects only the most aggressive part of the tumor rather than tumor heterogeneity. Recently, MTV and TLG have been identified as promising baseline prognostic factors in different lymphoma subtypes [39–42]. However, the outcomes of some studies that focused on DLBCL were inconsistent. One retrospective study indicated that high TLG values were independently predictive of reduced PFS and OS in DLBCL [43], whereas another retrospective study demonstrated that MTV was the only independent predictor of both PFS and OS; TLG did not predict PFS and was less predictive of OS than MTV [44]. Moreover, including metabolic heterogeneity and TLG, the simple prognostic model constructed by Ceriani et al. proves to be a predictor of outcome in primary mediastinal B-cell lymphoma [45]. However, Gormsen et al. highlighted the importance of nonstandardized clinical judgments and showed potential loss of valuable prognostic information when relying solely on semiautomated MTV measurements in a study of 118 patients of DLBCL [46]. In this study, we demonstrated that MTV was an independent predictor of OS but TLG seemed to be unrelated to survival outcome and that TLG was expected to be inferior to MTV due to the metabolic volume weighed by the SUV_mean_. Indeed, many physiological and technical factors might affect the computation of SUV. In contrast, MTV is not dependent on these factors as it is the result of processing a percentage of maximal uptake, irrespective of the unit of measurement [47]. The real utility of MTV and TLG in risk stratification and the possibility to combine TLG with other clinical or imaging parameters requires further exploration in the future.

The textural analysis is a process that extracts and analyzes quantitative imaging data from medical images to quantify the heterogeneous tumor microenvironment, which may be associated with the metabolic and pathological state of cancer [48, 49]. The term heterogeneity typically conveys different meanings depending on the imaging modality. Regarding PET, these parameters may be related to the cellular and molecular characteristics of the tumor such as fibrosis, hypoxia, receptor expression, and metabolism, while the low-dose CT refers to the variability in tissue density, which may result from the proportions of fat, air, and water [50–52]. Previous studies have confirmed the value of the texture parameters of ^18^F-FDG PET in the prediction of survival among patients with various types of cancer, including esophageal cancer, oropharyngeal cancer, and non-small-cell lung cancer [53, 54]. Some reports have demonstrated that CT-based texture analysis can potentially provide prognostic information [21–27]. However, no studies have evaluated the prognostic value of radiomics exploiting both ^18^F-FDG PET and low-dose CT (a component of PET-CT) in patients with PG-DLBCL to the best of our knowledge. Our results demonstrated that many of the texture parameters of ^18^F-FDG PET and low-dose CT were reliable indices in the prediction of the clinical outcomes of PG-DLBCL patients. However, quantification of heterogeneity using ^18^F-FDG PET/CT is still a relatively new methodology. Clinical markers and other metabolic baseline ^18^F-FDG PET/CT parameters were not found to be significant predictors of survival, probably because of the limited size of the study population.

**Figure 1:**
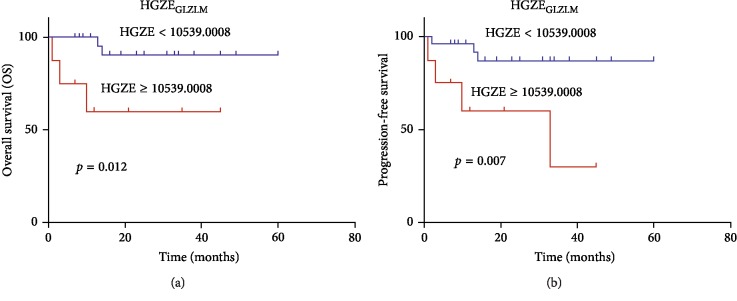
Kaplan–Meier plots of high gray-level zone emphasis from computed tomography scan. (a) Overall survival (OS). (b) Progression-free survival (PFS). Patients with high gray-level zone emphasis (HGZE) have poorer PFS and OS than those with low HGZE. GLZLM, gray-level zone-length matrix.

The use of PET/CT texture analysis in lymphoma patients is relatively scarce. Parvez et al. have regarded ^18^F-FDG PET uptake heterogeneity as a prognostic tool for aggressive B-cell lymphoma in a series of 82 patients. Several indices from the GLZLM were prognostic factors for disease-free survival, including LZE, LZLGE, and GLNU, while kurtosis was the only radiomic parameter correlated with OS [3]. Kurtosis, a histogram-based feature, reflects the shape of the gray-level distribution (peaked or flat) relative to a normal distribution and increases with higher heterogeneity. In this study, kurtosis was revealed to be a predictor of survival, which was similar to the finding of Parvez et al. In our study, univariate cox regression analysis revealed that GLNU was a significant predictor of OS and PFS. However, Orlhac et al. investigated the relationship among texture indices, SUV, MTV, and TLG, in three different tumor types and concluded that GLNU, correlated with tumor volume, was a surrogate of tumor volume and did not reflect the texture of the activity distribution [55]. Cox regression analysis indicated significant correlations between GLNU and tumor volume (Tables 7 and 8). Therefore, we used multivariate analysis to evaluate the prognostic values adjusted by tumor volume and concluded that both GLNU_GLZLM_ of CT and GLNU_GLRLM_ of PET were PFS predictors independent of tumor volume. Interestingly, HGZE_GLZLM_ turned out to be an outcome predictor associated with the PFS and OS of PG-DLBCL patients (Figure 1). This parameter measured the distribution of the high gray-level zones in the image, and there was a significant difference between the groups of patients dichotomized by the optimal cutoff, both for OS and PFS, with poorer survival in patients whose tumor had a higher HGZE_GLZLM_. Despite this promising finding, it is difficult to interpret the subtle differences in the meaning of the various heterogeneity parameters induced by different mathematical equations. Further investigation regarding the biological mechanisms of diverse heterogeneity parameters would be beneficial.

The current study has several limitations. Firstly, this was a retrospective study that might be affected by selection bias to a certain degree. Therefore, the results should be confirmed and validated in a further prospective study or by an external dataset. Secondly, the study cohort was relatively small, particularly for finding suitable parameters in texture analysis. The numbers of extracted features can be larger than that of the samples in a study, thus increasing the probability of overfitting the model, and the statistical significance has been corrected for multiple testing in the univariate analysis to avoid false discovery. As we have included all eligible patients in our institution, future studies should include data from other centers to validate our findings. Thirdly, the high reproducibility of the features is important in the development of clinical biomarkers. In our study, all images were acquired at the same center under the same acquisition method and reconstruction protocols, which mitigates the negative effects of reproducibility of radiomic features in PET/CT, particularly regarding geometric distortions. Furthermore, we should use more powerful statistical analyses, such as the machine learning domain neural network, support vector machine, and least absolute shrinkage and selection operator.

In conclusion, radiomic analysis of baseline ^18^F-FDG PET/CT indicated its potential for the prediction of outcomes in patients with PG-DLBCL, which may help us move towards individualized treatment. However, prospective studies with a large population are needed to validate the present findings.

## Figures and Tables

**Table 1:** Clinical characteristics of the patients.

Characteristics	Number of patients (%)
Age (years)	
Median	58
Range	26–79
≤58	18 (51.4)
>58	17 (48.6)
Sex	
Male	17 (48.6)
Female	18 (51.4)
Ann Arbor stage	
I	13 (37.1)
II	9 (25.7)
III	3 (8.6)
IV	10 (28.6)
IPI score	
0–1	24 (68.6)
2	4 (11.4)
3	5 (14.3)
4–5	2 (5.7)
LDH	
Cutoff	191 IU/L
<191 IU/L	23 (65.7)
≥191 IU/L	12 (34.3)
B Symptoms	
Yes	28 (80.0)
No	7 (20.0)

IPI, International Prognostic Index; LDH, lactate dehydrogenase.

**Table 2:** Radiomic parameters.

Index	Matrix	Parameter
Conventional indices		SUV_min_, SUV_mean_, SUV_max_, SUV_peak_, SUV_Std_
Advanced indices		MTV, TLG
Histogram-derived parameters		Skewness, kurtosis, entropy, energy
Shape-derived parameters		Sphericity, compacity

Texture features	GLCM	Homogeneity, energy, contrast, correlation, entropy, dissimilarity
	GLRLM	SRE/LRE, LGRE/HGRE, SRLGE/SRHGE, LRLGE/LRHGE, GLNU/RLNU, RP
	NGLDM	Coarseness, contrast, busyness
	GLZLM	SZE, LZE, LGZE, HGZE, SZLGE, SZHGE, LZLGE, LZHGE, GLNU, ZLNU, ZP

MTV: metabolic tumor volume; TLG: total lesion glycolysis; GLCM: gray-level cooccurrence matrix; GLRLM: gray-level run length matrix; SRE/LRE: short/long-run emphasis; LGRE/HGRE: low/high gray-level run emphasis; SRLGE/SRHGE: short run low/high gray-level emphasis; LRLGE/LRHGE: long-run low/high gray-level emphasis; GLNU/RLNU: gray-level nonuniformity/run length nonuniformity; RP: run percentage; NGLDM: neighborhood gray-level difference matrix; GLZLM: gray-level zone-length matrix; SZE/LZE: short/long-zone emphasis; LGZE/HGZE: low/high gray-level zone emphasis; SZLGE/SZHGE: short-zone low/high gray-level emphasis; LZLGE/LZHGE: long-zone low/high gray-level emphasis; GLNU/ZLNU: gray-level nonuniformity or zone-length nonuniformity; ZP; zone percentage.

**Table 3:** Univariate analysis (computed tomography).

	OS		PFS	
	HR (95%CI)	*p* value	HR (95%CI)	*p* value
B Symptom (yes vs. no)	0.257 (0.042–1.562)	0.140	0.213 (0.047–0.967)	**0.045** ^**∗**^

*Histogram based*				
Kurtosis (≥101.9046 vs.<101.9046)	8.399 (1.382–51.043)	**0.021** ^**∗**^	6.028 (1.091–33.300)	**0.039** ^**∗**^

*Shape based*				
Volume (≥47.5887 vs.<47.5887)	10.139 (1.131–90.883)	**0.038** ^**∗**^	6.426 (1.245–33.166)	**0.026** ^**∗**^
Sphericity (≥0.6407 vs.<0.6407)	6.679 (0.746–59.808)	0.090	10.157 (1.222–84.413)	**0.032** ^**∗**^

*GLRLM*				
HGRE (≥10445.0192 vs.<10445.0192)	6.222 (0.684–56.586)	0.105	9.216 (1.098–77.335)	**0.041** ^**∗**^
LRHGE (≥16855.0511 vs.<16855.0511)	8.867 (0.985–79.830)	0.052	5.603 (1.081–29.032)	**0.040** ^**∗**^
GLNU (≥880.6339 vs.<880.6339)	12.972 (1.448–116.210)	**0.022** ^**∗**^	8.049 (1.558–41.576)	**0.013** ^**∗**^
RLNU (≥4739.7637 vs.<4739.7637)	10.139 (1.131–90.883)	**0.038** ^**∗**^	6.426 (1.245–33.166)	**0.026** ^**∗**^

*GLZLM*				
LZE (≥3642.5187 vs.<3642.5187)	6.020 (1.003–36.139)	0.050	5.140 (1.143–23.106)	**0.033** ^**∗**^
HGZE (≥10539.0008 vs.<10539.0008)	7.330 (1.202–44.687)	**0.031** ^**∗**^	6.181 (1.365–27.976)	**0.018** ^**∗**^
LZLGE (≥0.4211 vs.<0.4211)	7.216 (1.190–43.764)	**0.032** ^**∗**^	6.284 (1.395–28.312)	**0.017** ^**∗**^
LZHGE (≥38755329.6111 vs.<38755329.6111)	6.020 (1.003–36.139)	0.050	5.140 (1.143–23.106)	**0.033** ^**∗**^
GLNU (≥90.9505 vs.<90.9505)	10.139 (1.131–90.883)	**0.038** ^**∗**^	6.426 (1.245–33.166)	**0.026** ^**∗**^
ZP (≥0.1439 vs.<0.1439)	0.227 (0.037–1.385)	0.108	0.195 (0.043–0.885)	**0.034** ^**∗**^

HGRE, high gray-level run emphasis; LRHGE, long-run high gray-level emphasis; GLNU, gray-level non-uniformity; RLNU, run length nonuniformity; LZE, long-zone emphasis; HGZE, high gray-level zone emphasis; LZLGE, long-zone low gray-level emphasis; GLRLM, gray-level run length matrix; GLZLM, gray-level zone-length matrix; LZHGE, long-zone high gray-level emphasis; ZP, zone length nonuniformity zone percentage; PFS, progression-free survival; OS, overall survival; HR, hazard ratio; CI, confidence interval. Asterisk (^*∗*^) indicates significance with a *p* value of <0.05 (shown in bold).

**Table 4:** Univariate analysis (positron emission tomography).

	OS		PFS	
	HR (95% CI)	*p* value	HR (95% CI)	*p* value
B Symptoms (yes vs. no)	0.257 (0.042–1.562)	0.140	0.213 (0.047–0.967)	**0.045** ^**∗**^
SUV_max_ (≥3.7173 vs. <3.7173)	34.057 (0.009–127920.296)	0.401	33.806 (0.031–36395.638)	0.323
MTV (≥66.5 vs. <66.5)	12.972 (1.448–116.210)	**0.022** ^**∗**^	8.049 (1.558–41.576)	**0.013** ^**∗**^
*Histogram based*				

Kurtosis (≥2.9179 vs. <2.9179)	13.090 (1.442–118.819)	**0.022** ^**∗**^	4.293 (0.945–19.509)	0.059
*Shape based*				

Volume (≥44.8928 vs. <44.8928)	10.139 (1.131–90.883)	**0.038** ^**∗**^	6.426 (1.245–33.166)	**0.026** ^**∗**^
Compacity (≥1.7565 vs. <1.7565)	116.242 (0.060–226261.65)	0.218	9.662 (1.161–80.383)	**0.036** ^**∗**^
*GLRLM*				

GLNU (≥178.7649 vs. <178.764)	10.968 (1.814–66.311)	**0.009** ^**∗**^	14.642 (2.661–80.549)	**0.002** ^**∗**^
RLNU (≥257.1264 vs. <257.126)	99.553 (0.060–164129.050)	0.224	8.487 (1.021–70.553)	**0.048** ^**∗**^

*NGLDM*				
Coarseness (≥0.0069 vs. <0.0069)	0.099 (0.011–0.884)	**0.038** ^**∗**^	0.156 (0.030–0.804)	**0.026** ^**∗**^

GLRLM, gray-level run length matrix; GLNU, gray-level nonuniformity; RLNU, run length nonuniformity; NGLDM, neighborhood gray-level difference matrix; SUV_max_, maximum standardized uptake value; MTV, metabolic tumor volume; LDH, lactate dehydrogenase; PFS, progression-free survival; OS, overall survival; HR, hazard ratio; CI, confidence interval. Asterisk (^*∗*^) indicates significance with a *p* value of <0.05 (shown in bold).

**Table 5:** Multivariate analysis (computed tomography).

	OS		PFS	
	HR (95%CI)	*p* value	HR (95%CI)	*p* value
*Histogram based*				
Kurtosis (≥101.9046 vs. <101.9046)	11.791 (1.583–87.808)	**0.016** ^**∗**^	—	—
*Shape based*				

Sphericity (≥0.6407 vs. <0.6407)	—	—	11.390 (1.360–95.371)	**0.025** ^**∗**^

*GLZLM*				
GLNU (≥90.9505 vs. <90.9505)	—	—	6.934 (1.069–44.981)	**0.042** ^**∗**^
HGZE (≥9917.8935 vs. <9917.8935)	9.805 (1.359–70.747)	**0.024** ^**∗**^	11.504 (1.921–68.888)	**0.007** ^**∗**^

GLNU, gray-level nonuniformity for zone; HGZE, high gray-level zone emphasis; GLZLM, gray-level zone length matrix; PFS, progression-free survival; OS, overall survival; HR, hazard ratio; CI, confidence interval. Asterisk (^*∗*^) indicates significance with a *p* value of <0.05 (shown in bold).

**Table 6:** Multivariate analysis (positron emission tomography).

	OS		PFS	
	HR (95%CI)	*p* value	HR (95%CI)	*p* value
*Histogram-based*				
MTV (≥66.5 vs. <66.5)	26.152 (2.089–327.392)	**0.011** ^**∗**^	—	—
Kurtosis (≥2.9179 vs. <2.9179)	28.685 (2.067–398.152)	**0.012** ^**∗**^	—	—

*GLRLM*				
GLNU (≥178.7649 vs. <178.7649)	—	—	14.642 (2.661–80.549)	**0.002** ^**∗**^

MTV, metabolic tumor volume; GLNU, gray-level nonuniformity; GLRLM, gray-level run length matrix; PFS, progression-free survival; OS, overall survival; HR, hazard ratio; CI, confidence interval. Asterisk (^*∗*^) indicates significance with a *p* value of <0.05 (shown in bold).

**Table 7:** Correlation between indices and volume (computed tomography).

Index	Volume
Kurtosis	0.606
GLNU_GLRLM_	0.981
RLNU_GLRLM_	0.992
LZE	0.849
HGZE	−0.366
LZLGE	0.848
LZHGE	0.851
GLNU_GLZLM_	0.953
ZP	−0.349

GLNU, gray-level nonuniformity; RLNU, run length nonuniformity; LZE, long-zone emphasis; HGZE, high gray-level zone emphasis; LZLGE, long-zone low gray-level emphasis; LZHGE, long-zone high gray-level emphasis; ZP, zone length nonuniformity zone percentage.

**Table 8:** Correlation between indices and volume (positron emission tomography).

Index	Volume
MTV	0.949
Kurtosis	0.222
Compacity	0.981
GLNU_GLRLM_	0.851
RLNU_GLRLM_	0.954
Coarseness	−0.911

MTV, metabolic tumor volume; GLNU, gray-level nonuniformity; RLNU, run length nonuniformity.

## Data Availability

The data used to support the findings of this study are included within the article.

## Additional Points

*Key Points*. Question: if texture parameters of PET/CT can predict the prognosis of primary gastric diffuse large B-cell lymphoma? Pertinent findings: in a cohort study indicating the potential of textural features for the prediction of outcomes in patients with PG-DLBCL in 35 patients underwent an FDG-PET/CT scan before treatment, many of the textural features extracted from both PET and CT datasets were significantly associated with OS and PFS. Implications for patient care: textural features extracted from both PET and CT datasets may help us move towards individualized treatment in PG-DLBCL and even in tumor.
